# Toward a Surrogate Marker of Malaria Exposure: Modeling Longitudinal Antibody Measurements under Outbreak Conditions

**DOI:** 10.1371/journal.pone.0021826

**Published:** 2011-07-27

**Authors:** Joseph J. Campo, Timothy J. Whitman, Daniel Freilich, Timothy H. Burgess, Gregory J. Martin, Denise L. Doolan

**Affiliations:** 1 Naval Medical Research Center, Silver Spring, Maryland, United States of America; 2 Barcelona Centre for International Health Research (CRESIB, Hospital Clínic-Universitat de Barcelona), Barcelona, Spain; 3 Infectious Diseases Department, National Naval Medical Center, Bethesda, Maryland, United States of America; 4 Division of Immunology, Queensland Institute of Medical Research, Brisbane, Queensland, Australia; 5 School of Medicine, University of Queensland, Brisbane, Queensland, Australia; New York University, United States of America

## Abstract

**Background:**

Biomarkers of exposure to *Plasmodium falciparum* would be a useful tool for the assessment of malaria burden and analysis of intervention and epidemiological studies. Antibodies to pre-erythrocytic antigens represent potential surrogates of exposure.

**Methods and Findings:**

In an outbreak cohort of U.S. Marines deployed to Liberia, we modeled pre- and post-deployment IgG against *P. falciparum* sporozoites by immunofluorescence antibody test, and both IgG and IgM against the *P. falciparum* circumsporozoite protein by enzyme-linked immunosorbant assay. Modeling seroconversion thresholds by a fixed ratio, linear regression or nonlinear regression produced sensitivity for identification of exposed U.S. Marines between 58–70% and specificities between 87–97%, compared with malaria-naïve U.S. volunteers. Exposure was predicted in 30–45% of the cohort.

**Conclusion:**

Each of the three models tested has merits in different studies, but further development and validation in endemic populations is required. Overall, these models provide support for an antibody-based surrogate marker of exposure to malaria.

## Introduction

Assessment of malaria burden is critical for the evaluation of malaria control measures. We currently lack tools for discrimination of host exposure to *Plasmodium falciparum* parasites. In assessment of interventions, correct classification of “immune” and “unexposed” cohorts is important for interpretation of results [1]. Biomarkers of exposure could mitigate classification error and facilitate clinical trial designs [2].

Antibody responses to blood stage parasites have been effectively modeled as a tool for estimating transmission intensity in endemic populations [3], [4] and support the use of antibodies as biomarkers of exposure. However, these models do not discriminate exposure to parasite inoculum (sporozoites) from blood stage parasites, the former being especially applicable to assessment of interventions that reduce or prevent blood stage infections or parasite transmission. Antibody responses to pre-erythrocytic (sporozoite/liver) antigens represent potential markers of exposure. These antigens have been shown to reflect exposure across varying transmission intensities [5], and travelers to endemic areas often show high levels of sporozoite-specific antibodies [6]. In particular, the circumsporozoite protein (CSP) is an ideal target due to its pronounced expression from point of inoculation to residency in hepatocytes [7]. In a recent trial of experimental infections in humans, 80% of naïve volunteers inoculated with sporozoites seroconverted with antibodies against sporozoite antigens, in particular against CSP [8]. However, the broad use of CSP as a biomarker may be limited by the variability in CSP-specific antibody reactivity following exposure in children [9], and across age groups and transmission settings [10]. Low prevalence antibodies to CSP in areas of unstable transmission suggest that persistent antigen exposure is required to maintain antibody levels [11]. Indeed, recent studies show that B cell memory to malaria antigens is slowly produced and wanes without re-exposure [12]. Antibody half-life following acute infection varies from a couple of weeks to several months, but generally decay rapidly [13]. Antibody decay has been observed to be notably faster in very young children compared to older children, perhaps due to intrinsic differences in the generation of short-lived and long-lived plasma cells with age [14]. In infants, short peaks of antibody responses to the blood stage antigen MSP-1 observed during the first year of life did not appear to be maintained at higher post-infection levels than pre-infection [15]. Conversely, a study in Thailand showed that malaria-specific B-cell memory and antibody production may persist for years following infection [16]. Interestingly, antibody prevalence in a low-transmission region of Peru persisted through the 4-month non-transmission season, although it was noted that children responded more slowly than adults [17]. These findings demonstrate B cell memory capacity may be both age-dependent and influenced by exposure. In Mali, a population of memory B cells expressing inhibitory receptors and responding poorly to mitogen stimulation was expanded in individuals with chronic parasite exposure [18]. “Atypical” memory B cells were also observed at lower levels in Peru and correlated with the lower transmission intensity [19]. Neither the function or causal association with malaria has been established for atypical B cells, but they may be indicative of the suboptimal antibody generation and maintenance observed in areas of high malaria transmission. These observations suggest that generation and decay of immunological memory is subject to a highly complex immunoepidemiology.

To date, there is no surrogate marker of exposure to the bite of infected mosquitoes, and modeling data that reflect natural exposure is difficult. Henceforth, infection and exposure refer to inoculum of sporozoites from the bite of infected mosquitoes, regardless of resulting blood stage infection. In this study, we examine longitudinal antibody responses during a high incidence outbreak of *P. falciparum* malaria among U.S. Marines deployed to Liberia from August to October, 2003 [20]. Although the exact level of exposure is undefined, this cohort of Marines provides a unique opportunity to study antibody acquisition following exposure. Another study of military personnel after deployment to a malaria endemic area reported high antibody prevalence to pre-erythrocytic antigens that demonstrated exposure [21]. In this study, we aimed to describe antibody responses to the immunodominant sporozoite surface antigen, the CSP, and to the whole sporozoite, and to propose longitudinal models for a surrogate marker of exposure.

## Methods

### Ethics Statement

Coded de-identified plasma samples were assayed for CSP- or sporozoite-specific antibodies under a protocol approved by the Naval Medical Research Center (NMRC) Institutional Review Board (IRB) with a specific waiver from the IRB for the requirement for informed consent (protocol#NMRC.2005.0003). That same protocol approved the use of coded de-identified plasma samples from malaria-naïve individuals collected with written informed consent in support of various IRB-approved NMRC clinical studies. All procedures reported herein were reviewed and approved by the NMRC IRB and all research was conducted in compliance with all applicable Federal Regulations governing protection of human subjects.

### Samples

A total of 330 paired pre-deployment and post-deployment sera were obtained from the Department of Defense Serum Repository (White Oak, MD) corresponding to 165 of the 225 Marines of the 26^th^ Marine Expeditionary Unit (26^th^ MEU) deployed in a holoendemic area of Liberia for 10–12 days [20]. Pre-deployment sera were collected as part of routine health care between November 2000 and May 2003. Post-deployment sera were collected during the outbreak investigation between September 2003 and February 2004. Results for thick film blood slides were available for hospitalized Marines [20].

Plasmas from 42 naïve U.S. volunteers were used as the negative comparator group and as a confirmed “unexposed” group.

### Indirect fluorescence antibody test (IFAT)

The Indirect fluorescence antibody test (IFAT) was used to detect antibodies against *P. falciparum* sporozoites. NF54 strain sporozoites were isolated from infected *Anopheles stephensi* mosquitoes by salivary gland dissection. Sporozoites were coated on 12-well HTC Super Cured glass slides (Cel-Line Associates, Inc., Newfield, NJ) at approximately 5,000 sporozoites per well, dried in an air-locked box with desiccant and stored at −70°C. Slide wells were blocked with 1% BSA (Sigma, St. Louis, MO). Sera were applied in a 12-step, 2-fold dilution series beginning at a dilution factor of 20, and incubated for 1 hour at 37°C. Slides were washed 3 times with PBS. Slide wells were stained with FITC-labeled anti-human IgG (MP Biomedicals, Solon, OH) diluted 1∶100 in 0.05% Evans Blue-PBS and incubated at 37°C for 30 minutes. Slides were washed as before and covered with Vectashield mounting medium (Vector Laboratories, Inc., Burlingame, CA). Endpoint titer was scored by fluorescence microscopy, corresponding to the lowest dilution at which sporozoite fluorescence was observed. On each assay day, anti-PfCSP monoclonal antibody NFS1 was assayed in parallel as a positive control, using FITC-labeled anti-mouse IgG (Beckton Dickenson, Franklin, NJ). The positive control was not used for data adjustment or assay normalization. All paired samples were read on the same day with the same batch of coated slides.

### ELISA

Enzyme-linked immunosorbant assay (ELISA) was used to detect antibodies against recombinant *P. falciparum* CSP (residues 19–405). This capture antigen, produced in *Escherichia coli*, has been previously described [22]. Immunolon II microtiter plates (Dynatech Laboratory Inc., Chantilly, VA) were coated with 25 ng recombinant CSP (3D7 strain) per well and blocked with 5% nonfat dry milk (NFDM) in PBS. Sera were assayed at dilutions of 1/50, 1/100, 1/200 and 1/400. Data are reported for the 1/100 dilution, as this was the highest dilution with low background. Plates were incubated with 50 µL/well HRP-labeled anti-human IgG or IgM (Kirkegaard & Perry Laboratories, Gaithersburg, MD) diluted 1∶2000 in 3% NFDM, and developed using ABTS substrate system (Kirkegaard & Perry Laboratories, Gaithersburg, MD). Optical densities (OD) were recorded on a SpectraMax 190 spectrophotometer (Molecular Devices, Silicon Valley, CA) at 405 nm. NFS1 monoclonal antibody was used as a positive control for the assay, but not for data adjustment. No inter-assay normalization procedures or data adjustments were employed, except removal of background OD from black wells, since the primary comparison was ratio of post-exposure to pre-exposure read-outs. Hence, paired samples were assayed on the same ELISA plates to avoid inter-assay variation.

### Exposure models

Three exposure models were tested. For a “fixed ratio” model, a threshold of a post-/pre-deployment OD or endpoint titer ratio of 2 was used to define seroconversion. A receiver-operator characteristics (ROC) curve was constructed to test sensitivity and specificity of variable fixed ratios. Area under the curve (AUC) demonstrates trade-off between sensitivity and specificity; an AUC of 1.0 is considered a perfect diagnostic model. A linear regression model of post- vs. pre-deployment OD used ordinary least squares (OLS) regression on data transformed using the natural logarithm, and the lower bound of the 95% confidence interval of the regression was used as the seroconversion threshold. A non-linear regression model of non-transformed OD ratios with pre-deployment ODs used an exponential decay equation,
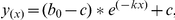
where *b_0_* is the initial OD ratio, *k* is the “decay rate” and *c* is a constant corresponding to the asymptote of the decay. As before, the lower bound of the 95% confidence interval of the regression line was used as the seroconversion threshold. Data demonstrating inconstant variance across the range of measurements (heteroskedasticity) were re-fit using robust regression models, which are less sensitive to violations in the assumption on constant variance. Individuals demonstrating IgG and/or IgM seroconversion were considered “exposed” according to each model.

### Data analysis

Databases for both ELISA and IFAT data were managed in Microsoft Excel 2003 and analyzed in STATA version 11 (StataCorp, LP, College Station, TX). IFAT and IgG ELISA correlation was calculated using Pearson's correlation coefficient (ρ). Agreement between IFAT and ELISA and between exposure models was assessed using Cohen's kappa score. Sensitivity analysis was performed on each model for discrimination between exposed (hospitalized Marines) and non-exposed (naïve volunteers) or between confirmed and suspected exposures by calculating sensitivity, specificity, positive and negative predictive power and likelihood ratios with 95% confidence intervals. Difference between sensitivity and specificity of models was tested using McNemar's exact test, stratified by exposure group. Statistical significance was considered for p<0.05.

## Results

**Figure 1:**
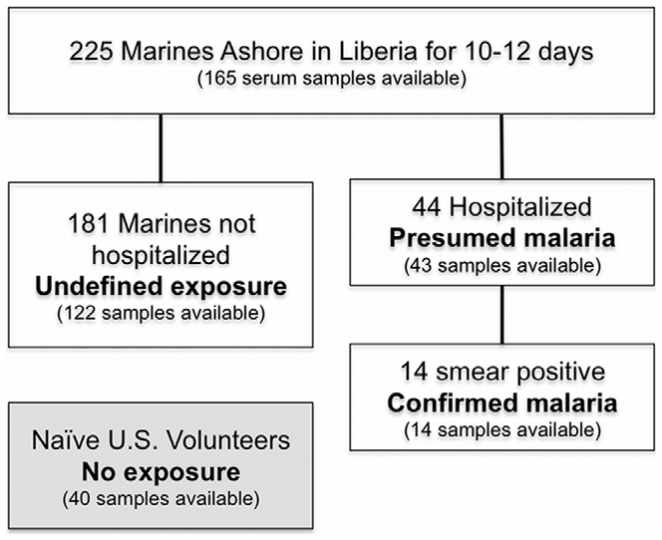
Patient group and sampling flowchart. Of the 225 Marines of the 26^th^ Marine Expeditionary Unit sent ashort to Liberia in August, 2003, 165 serum samples were collected both prior to and after a high incidence malaria outbreak. Of these Marines, 44 were hospitalized with presumed malaria, of which 14 were confirmed positive by blood smear. The non-hospitalized Marines were considered as a cohort with undefined exposure, do to the confounding factor of mefloquine prophylaxis or early preventive treatment. 40 naïve U.S. adult volunteers comprised the unexposed comparator group (grey box).

During the outbreak investigation, 80/225 (35.6%) of the 26^th^ MEU were diagnosed with presumed *P. falciparum* malaria; 44 Marines were airlift evacuated and hospitalized, whilst 36 Marines with febrile illness were managed shipboard for illness [20]. Of the 44 hospitalized Marines with presumed malaria, 14 (32%) had *P. falciparum* positive blood slides (Figure 1).

**Figure 2:**
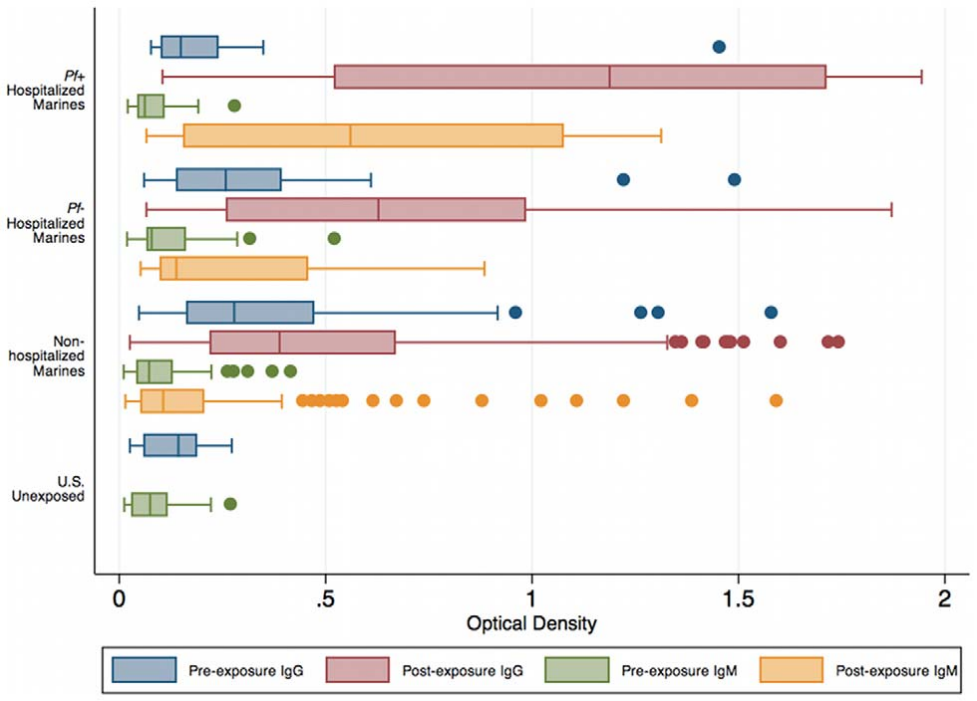
Range of optical density measurements in the 26^th^ MEU and U.S. volunteers. The boxplot represents ranges of optical density readings in pre- and post-deployment sera. *P. falciparum* positive (*Pf*+) and *Pf*− hospitalized Marines represent the confirmed and presumed exposure groups, respectively. Boxes are median and interquartile ranges, whiskers include outside values and dots represent outliers.

A seropositivity threshold was defined as the mean plus three standard deviations of the malaria-naïve comparator group (U.S. non-exposed volunteers). This criterion was considered conservative, since the threshold is the upper limit of the 99.7% confidence interval of the negative controls, based on a normal distribution (Shapiro-Wilk W test for normality, p = 0.07). None of the negative controls had detectable fluorescence in the IFAT, and all test samples which scored positive at any dilution of the IFAT were considered seropositive. In total, 158 Marines were assayed by sporozoite IFAT and 165 by ELISA for IgG and IgM. Ranges of optical density readings by ELISA are shown in Figure 2. The mean IFAT titer was 8.78 (95% CI 5.93–11.62) before deployment and 35.27 (95% CI 21.68–48.85) post-deployment. IFAT and IgG ELISA showed poor correlation (pre-deployment ρ = 0.24; post-deployment ρ = 0.59). Seropositivity in the pre-deployment sera was considered evidence of exposure before the outbreak; 37/158 (22%) exhibited evidence of previous exposure by IFAT and 64/165 (39%) by IgG ELISA, 5 of which were also positive for IgM. Only one individual was positive for IgM only.

**Figure 3:**
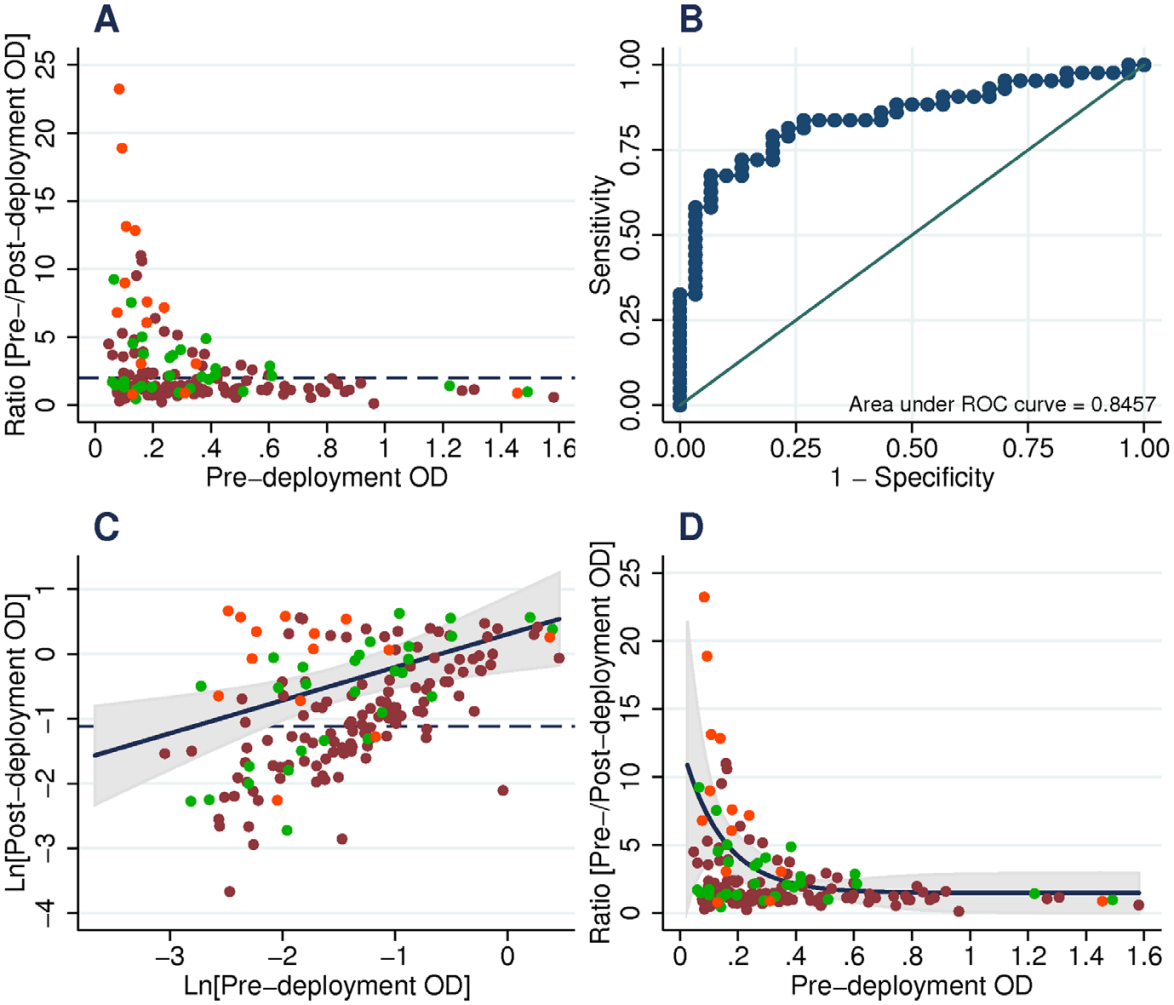
anti-CSP IgG seroconversion models applied to the 26^th^ MEU. *P. falciparum* smear positive hospitalized Marines (orange dots), *P. falciparum* smear negative hospitalized Marines (green dots) and remainder of 26^th^ MEU (maroon dots). Fixed ratio model *(A)* seroconversion threshold is post-/pre-deployment ratio of 2 (solid line). The ROC curve *(B)* shows sensitivity vs specificity of the fixed ratio model using seroconversion of hospitalized Marines (exposed) against naïve U.S. volunteers (unexposed). Linear regression model *(C)* uses an ordinary least squares regression line on all hospitalized marines (orange and green dots); 95% CI limits are shown in grey, positive OD threshold is shown (dashed line) and seroconversion threshold is lower bounds of 95% CI limits. The exponential decay model *(D)* uses a non-linear negative exponential regression line on all hospitalized Marines (orange and green dots); 95% CI limits are shown in gray and seroconversion threshold is lower 95% CI limits. Data for IgM ELISA and sporozoite IFAT are not shown.

In the fixed ratio model (Figure 3A), seroconversion prevalence was 49/158 (31%) by IFAT, 50/165 (30%) by IgG ELISA, 20 of which also seroconverted for IgM, and 15/165 (9%) by IgM ELISA only. A ROC curve was constructed for each test using naïve U.S. volunteers as the “non-exposed” group and hospitalized Marines as the “exposed” group (Figure 3B). AUC was 0.68 (95% CI 0.57–0.80) for IFAT, 0.85 (95% CI 0.76–0.94) for IgG ELISA and 0.89 (95% CI 0.81–0.97) for IgM ELISA. The fixed ratio model predicted that 56/158 (35%) of Marines were exposed by IFAT and 65/158 (39%) by ELISA. Agreement between IFAT and IgG ELISA was 84% (kappa score: 0.62, p<0.0001).

To account for pre-existing antibody on antibody reactivity following exposure, regression models were used to establish dynamic seroconversion thresholds. Linear regression models (Figure 3C) returned seroconversion prevalence of 37/165 (22%) IgG only, 33 (20%) both IgG and IgM, and 8/165 (5%) IgM only. Prediction of exposure in the 26^th^ MEU was 74/165 (45%). To control for the magnitude of change between pre- and post-deployment antibodies, nonlinear regression on OD ratios vs. pre-deployment OD was fitted (Figure 3D). Seroconversion prevalence was 34/165 (21%) IgG only, 31/165 (19%) both IgG and IgM, and 9/165 (5%) IgM only. Exposure was predicted in 74/165 (45%) of the 26^th^ MEU.

Agreement between the two regression models was 95% (kappa score: 0.90, p<0.0001), and agreement between the fixed ratio model and linear or nonlinear regression was 87% (kappa score: 0.74, p<0.0001) and 92% (kappa score: 0.84, p<0.0001), respectively. The ability of each model to discriminate between individuals with presumed exposure or none was similar, with overlapping confidence intervals in all measurements of sensitivity, specificity, positive and negative predictive values, and likelihood ratios (Table 1), and no method showed difference in proportion of exposures (marginal homogeneity, exact p>0.05). All models showed lower specificity when discriminating between hospitalized Marines with positive or negative blood smears. No method showed significant differences in this comparison (marginal homogeneity, exact p>0.05).

**Table 1:** Diagnostic sensitivity analysis of exposure models by comparison group.

Model	Sensitivity (95% CI)	Specificity (95% CI)	PPV (95% CI)	NPV (95% CI)	LR+^b^ (95% CI)	LR−b (95% CI)
*26^th^ MEU with presumed exposure* a *vs. naïve U.S. volunteers*						
Fixed ratio: IFAT	58.1 (42.1–73.0)	90.6 (75.0–98.0)	89.3 (71.8–97.7)	61.7 (46.4–75.5)	6.2 (2.1–18.8)	0.5 (0.3–0.7)
Fixed ratio: ELISA	67.4 (51.5–80.9)	87.5 (71.0–96.5)	87.9 (71.8–96.6)	66.7 (50.5–80.4)	5.0 (2.1–13.8)	0.4 (0.2–0.6)
Linear regression	69.8 (53.9–82.8)	96.9 (83.8–99.9)	96.8 (83.3–99.9)	70.5 (54.8–83.2)	22.3 (3.2–155.2)	0.3 (0.2–0.5)
Nonlinear regression^b^	67.4 (51.5–80.9)	96.9 (83.8–99.9)	96.7 (82.8–99.9)	68.9 (53.4–81.8)	14.8 (3.1–71.4)	0.4 (0.2–0.5)
*26^th^ MEU with confirmed exposure (P. falciparum smear positive) vs. presumed exposure (P. falciparum smear negative)*						
Fixed ratio: IFAT	64.3 (35.1–87.2)	44.8 (26.4–64.3)	36.0 (18.0–57.5)	72.2 (46.5–90.3)	1.2 (0.7–1.9)	0.8 (0.4–1.8)
Fixed ratio: ELISA	85.7 (57.2–98.2)	41.4 (23.5–61.1)	41.4 (23.5–61.1)	85.7 (57.2–98.2)	1.5 (1.0–2.1)	0.35 (0.1–1.3)
Linear regression	85.7 (57.2–98.2)	37.9 (20.7–57.7)	40.0 (22.7–59.4)	84.6 (54.6–98.1)	1.4 (1.0–2.0)	0.4 (0.1–1.5)
Nonlinear regression^b^	78.6 (49.2–95.3)	37.9 (20.7–57.7)	37.9 (20.7–57.7)	78.6 (49.2–95.3)	1.2 (0.8–1.9)	0.6 (0.2–1.7)

**NOTE.** IFAT: immunofluorescence assay test; ELISA: enzyme-linked immunosorbent assay; PPV: positive predictive power; NPV: negative predictive power; LR: likelihood ratio.

a 43 hospitalized Marines diagnosed with *P. falciparum* malaria according to clinical criteria.

b Likelihood ratios estimated using the substitution formula (0.5 added to all 2×2 table frequencies).

## Discussion

An outbreak of *P. falciparum* malaria among U.S. Marines provided an opportunity to investigate a surrogate marker of exposure in a cohort with variable exposure history. Indeed, many of the 26^th^ MEU were seropositive to CSP prior to deployment in Liberia, unsurprising, since many of them were likely deployed previously in malaria endemic areas. Adherence to chemoprophylaxis provided before deployment, among other preventive measures, was believed to be low, although adherence could be a confounding factor in assessment of exposure. Only a third of the hospitalized Marines gave a positive blood slide, which may be explained by antimalarial therapy of febrile Marines before definitive laboratory testing [20]. Regardless, the clinical characteristics of the outbreak lead us to suspect that the entire group of hospitalized Marines was exposed to malaria.

Multiple models were tested to find the most suitable for a surrogate marker of exposure. However, the data from this cohort do not highlight any superior model. The fixed ratio model is practical, but may be best applied to travelers or those with little history of exposure, as antibody responses in pre-exposed individuals increase less dramatically after exposure. Indeed, sensitivity of the fixed ratio model seemed slightly higher for the IFAT when comparing only those marines without evidence of pre-exposure against controls (sensitivity: 70.0%, 95% CI 50.6–85.3%), although the sensitivity by ELISA did not change. For studies in malaria endemic populations, a regression model that controls for baseline antibodies would be more appropriate. The models employed here must be tested in populations under different exposure patterns. A limitation of these data is the large range of time between outbreak and blood sample collection. Given short antibody half-lives, detection may have been dampened by loss of reactivity [13]. Additionally, some individuals with confirmed exposure had no anti-CSP antibody response. The most severe malaria case showed no antibodies to CSP. This is consistent with other studies that have noted absent antibody responses following severe malaria infection [23]. Inclusion of additional antigens to control for heterogeneity in antibody responsiveness may increase the robustness of such models.

These models may help in distinguishing protection from lack of exposure in assessment of interventions. The high positive predictive value observed in these data suggests that the “exposures” were correctly classified. Improvements to the models must be made before “unexposed” groups may be classified due to lower negative predictive values (Table 1). Data from additional pre-erythrocytic antigens should be fitted as well, since the utility of CSP may be compromised if a licensed vaccine such as RTS,S is employed [24], and the disagreement between ELISA and IFAT suggests that additional sporozoite antigens may be present.

While these exposure models may not directly apply to other data sets, these data lay groundwork for subsequent studies of surrogate markers of exposure. The complexity of the immune response to malaria warrants further modeling of antibodies, and we plan to continue development of these models by testing them in well-characterized malaria endemic populations and using additional antigens, including commercially produced peptides. It is likely that important variables are missing from the regression models, such as age and time since exposure. Future modeling exercises should attempt to control for these variables.
